# The Cavernous Nerve Injury Rat Model: A Pictorial Essay on Post-Radical Prostatectomy Erectile Dysfunction Research

**DOI:** 10.3390/life13122337

**Published:** 2023-12-13

**Authors:** Silviu Constantin Latcu, Dorin Novacescu, Victor-Bogdan Buciu, Cristina-Stefania Dumitru, Raluca Amalia Ceausu, Marius Raica, Talida Georgiana Cut, Razvan Ilina, Daniel Claudiu Malita, Cristi Tarta, Alin Adrian Cumpanas

**Affiliations:** 1 Doctoral School, Victor Babes University of Medicine and Pharmacy Timisoara, E. Murgu Square, No. 2, 300041 Timisoara, Romania; silviu.latcu@umft.ro (S.C.L.); victor.buciu@umft.ro (V.-B.B.); talida.cut@umft.ro (T.G.C.); 2 Department XV, Discipline of Urology, Victor Babes University of Medicine and Pharmacy Timisoara, E. Murgu Square, No. 2, 300041 Timisoara, Romania; cumpanas.alin@umft.ro; 3 Department II, Discipline of Histology, Victor Babes University of Medicine and Pharmacy Timisoara, E. Murgu Square, No. 2, 300041 Timisoara, Romania; cristina-stefania.dumitru@umft.ro (C.-S.D.); ra.ceausu@umft.ro (R.A.C.); marius.raica@umft.ro (M.R.); 4 Angiogenesis Research Center, Victor Babes University of Medicine and Pharmacy Timisoara, E. Murgu Square, No. 2, 300041 Timisoara, Romania; 5 Department XIII, Discipline of Infectious Diseases, Victor Babes University of Medicine and Pharmacy Timisoara, E. Murgu Square, No. 2, 300041 Timisoara, Romania; 6 Center for Ethics in Human Genetic Identifications, Victor Babes University of Medicine and Pharmacy Timisoara, E. Murgu Square, No. 2, 300041 Timisoara, Romania; 7 Department IX, Discipline of Surgical Semiology II, Victor Babes University of Medicine and Pharmacy Timisoara, E. Murgu Square, No. 2, 300041 Timisoara, Romania; razvan.ilina@umft.ro; 8 Department XV, Discipline of Radiology and Medical Imaging, Victor Babes University of Medicine and Pharmacy Timisoara, E. Murgu Square, No. 2, 300041 Timisoara, Romania; malita.daniel@umft.ro; 9 Department X, Discipline of General Surgery II, Victor Babes University of Medicine and Pharmacy Timisoara, E. Murgu Square, No. 2, 300041 Timisoara, Romania; tarta.cristi@umft.ro

**Keywords:** post-radical prostatectomy erectile dysfunction, prostate cancer treatment-associated morbidity, cavernous nerve injury rat model, animal model standardization, illustrative surgical protocols, mean arterial pressure, intra-cavernosal pressure, comparative pelvic anatomy between rats and humans, cavernous nerve dissection, injury and electrostimulation

## Abstract

Understanding and addressing post-radical prostatectomy (RP) erectile dysfunction (ED) is of paramount importance for clinicians. Cavernous nerve (CN) injury rat model studies have provided consistently promising experimental data regarding regaining erectile function (EF) after nerve damage-induced ED. However, these findings have failed to translate efficiently into clinical practice, with post-RP ED therapeutic management remaining cumbersome and enigmatic. This disparity highlights the need for further standardization and optimization of the elaborate surgical preparation protocols and multifaceted reporting parameters involved in reliable CN injury rat model experimentation. Even so, despite its technical complexity, this animal model remains instrumental in exploring the functional implications of RP, i.e., surgical lesions of the neurovascular bundles (NVBs). Herein, besides cavernous nerve (CN) dissection, injury, and electrostimulation, multiple pressure measurements, i.e., mean arterial pressure (MAP) and intra-cavernosal pressure (ICP), must also be achieved. A transverse cervical incision allows for carotid artery cannulation and MAP measurements. Conversely, ICP measurements entail circumcising the penis, exposing the ischiocavernous muscle, and inserting a needle into the corporal body. Finally, using an abdominal incision, the prostate is revealed, and the major pelvic ganglia (MPG) and CNs are dissected bilaterally. Specific surgical techniques are used to induce CN injuries. Herein, we provide a narrative and illustrative overview regarding these complex experimental procedures and their particular requirements, reflecting on current evidence and future research perspectives.

## 1. Introduction

Erectile dysfunction (ED), a prevalent medical condition, afflicts a substantial portion of men aged 40 to 70, with an estimated incidence of up to 52% [1]. This condition has exhibited a rising trend in recent decades, potentially affecting a staggering 150 million men worldwide [2]. The pathophysiology of ED encompasses various factors, including vasculogenic, neurogenic, anatomical, hormonal, drug-induced, and psychogenic etiologies [3]. Notably, radical prostatectomy stands out as a common iatrogenic contributor to ED, with reported postoperative rates ranging from 14% to 90% [4].

The introduction of prostate-specific antigen (PSA) screening, alongside the emergence of novel diagnostic imaging tools [5], has ushered in a new era of early prostate cancer (PCa) detection. Consequently, an increasing number of patients are expected to experience favorable long-term oncologic outcomes following radical prostatectomy (RP). However, in contrast to this positive trend, research has revealed that only 23% of men under the age of 60 regain their complete erectile function (EF) after undergoing bilateral nerve-sparing RP (nsRP) [6]. In the context of nerve-sparing surgery, ED emerges as a consequence of iatrogenic manipulation damage to the periprostatic neurovascular bundles (NVBs), involving predominantly neuropraxia of the cavernous nerves (CNs) [7].

Neurapraxia, as per the Seddon classification, denotes a temporary disruption in nerve conduction due to damage to the myelin sheath. This temporary CN conduction block results in a decrease in the frequency and quality of both daily and nocturnal erections, contributing to persistent cavernous hypoxia [8]. Both in vitro and in vivo studies have suggested that neurapraxia and the subsequent penile hypoxia may lead to collagen accumulation, smooth muscle apoptosis, and fibrosis [9].

Ultimately, these alterations in penile tissue pave the way for venous leakage and permanent ED to occur, even before complete restoration of nerve integrity can be achieved, which can extend up to 2 years post-surgery [10]. It is noteworthy that ED following RP differs from classical vasculogenic ED [11]. In human corpus cavernosum tissue, endothelial function tends to remain preserved in patients after RP, whereas significant disruptions are observed in neurogenic relaxation, characterized by sympathetic hyperinnervation.

Phosphodiesterase type 5 (PDE5i) therapy serves as the primary pharmacological intervention for ED. However, it is vital to acknowledge that PDE5i treatment is associated with a failure rate of approximately 35%, thus necessitating further investigation, especially as a gold standard treatment, in this rapidly expanding urological field of post-RP ED clinical management [12]. Recent scientific endeavors have delved into the exploration of autologous stem cell transplantation as an innovative alternative approach [13].

A substantial proportion of our current understanding of the pathophysiology of erections and the development of treatment strategies has been derived from the utilization of animal models. In the period spanning from the 1960s to the 1990s, larger animals such as cats, dogs, and monkeys were employed for the purpose of analyzing erectile responses, post-CN injury, or in a systemic context (e.g., diabetes, atherosclerosis, etc.). However, since the introduction of the rat model for investigating penile erection by Quinlan et al., rodents have predominantly supplanted other species as experimental applications in this research field. Quinlan et al. initially demonstrated that electrical stimulation of the cavernous nerve in Sprague Dawley rats consistently induced tumescence of the corpus cavernosum. Subsequently, this rat model has been widely adopted and refined [14].

It is important to note that the rat model, which involves electrostimulation of the CN and the measurement of both the pressure within the cavernous bodies of the penis, i.e., intra-cavernosal pressure (ICP), and the systemic mean arterial pressure (MAP) for reference, presents technical challenges and can be daunting for research teams lacking expertise in this specialized technique. With these challenges in mind, we designed the current pictorial essay and narrative review of key medical literature, focusing on the primary objective of providing a comprehensive practical guideline tailored to novice bench researchers embarking on studies using Sprague Dawley rats as the primary model for experimentation, with a specific focus on its potential clinical implications, in the context of post-RP ED. Ultimately, our paper aims to provide a starting point for possible advancement in this area of bench research by identifying the strengths and potential shortcomings of these experimental approaches.

## 2. Comparative Pelvic Anatomy and Experimental Design Considerations

**Figure 1:**
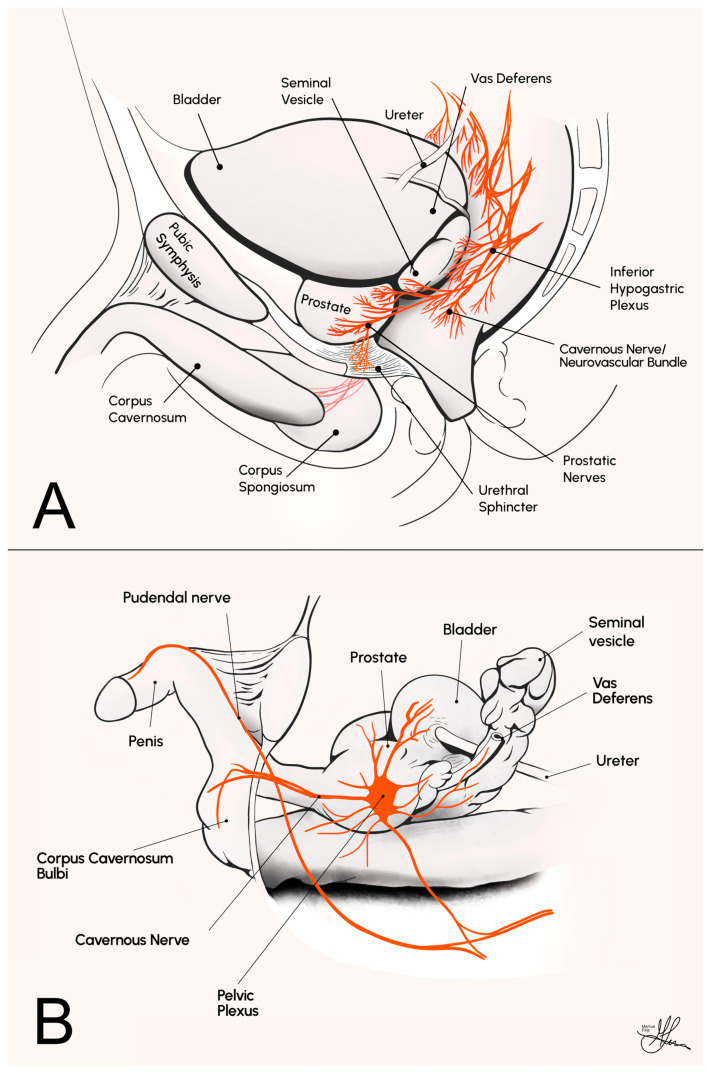
Comparative anatomy illustrations of the male pelvic cavity, sagittal view: (**A**) in humans; (**B**) in rats [23].

The CNs are a pair of parasympathetic nerves originating from the inferior hypogastric (or pelvic) plexus. They course along the lateral aspect of the prostate, i.e., adopting a plexus anatomic conformation and constituting the periprostatic NVBs in humans (see Figure 1A), before entering the corpus cavernosum at the base of the penis. Their primary role involves regulating blood flow into the erectile tissue, and they are pivotal in the physiological process of sexual arousal and penile erection [15].

To further elaborate, in the physiological context, erection initiation stems from the pelvic plexus, with cavernous nerves (CNs) containing both parasympathetic and sympathetic fibers. These CNs transition from a mix of myelinated and unmyelinated fibers to predominantly unmyelinated axons at the crural entry, crucial for penile innervation, particularly nitric oxide (NO) release [16,17]. Thus, these fibers are responsible for controlling the tonus of the smooth muscle within the penile blood vessels [18]. As a result of their regulatory function, the CNs have the capacity to increase blood flow to the corpus cavernosum, ultimately leading to the occurrence of penile erection. Consequently, any significant damage to the CNs can result in ED [4].

Herein, irrespective of the level of surgical expertise or the specific surgical methodology employed, RP invariably inflicts some degree of NVB/CN injury [19]. This unavoidable neural trauma can emanate from various intraoperative factors, including retraction-induced mechanical injury, electrocautery-induced thermal damage, disruption of neural vasculature, or subsequent localized inflammation following compression trauma [20]. This surgically induced neural insult serves as the etiological factor underlying the impairment of parasympathetic-mediated penile function, clinically manifesting as post-RP ED [21].

From a physiological perspective, penile erection involves the relaxation of smooth muscle in the penis, primarily through the L-arginine–nitric oxide (NO)–guanylyl cyclase–cyclic guanosine monophosphate (cGMP) pathway. Sexual arousal stimulates the release of NO, which enters smooth muscle cells and activates guanylyl cyclase. This enzyme converts guanosine triphosphate to cGMP, the key intracellular trigger for erection. cGMP activates protein kinase G (PKG), leading to decreased intracellular calcium levels and relaxation of arterial and trabecular smooth muscle, resulting in penile erection. Phosphodiesterase type 5 (PDE5) regulates this process by degrading cGMP. PDE5 inhibitors, like sildenafil, enhance erection by competing with cGMP for PDE5, thereby increasing cGMP levels. These inhibitors amplify the normal cGMP-dependent relaxation mechanisms in the presence of NO but are ineffective without NO stimulation. This mechanism highlights the balance between NO production and PDE5 activity in regulating penile erection [22].

Extensive research findings suggest that the regulatory mechanisms governing penile erection exhibit significant physiological, biochemical, and pharmacological similarities among various mammalian species, including humans [24]. For instance, when corpus cavernosum tissue from humans, monkeys, rabbits, dogs, rats, and mice is precontracted to simulate penile flaccidity, it responds similarly to neural and endothelial signals, eliciting relevant NO-dependent relaxant responses that mimic the process of erection [25,26]. Consequently, a critical role for the NO pathway in penile erection has been established through in vivo studies involving various animal models [27]. Among these species, Sprague Dawley rats are the most commonly used for in vivo studies on penile erection, usually at around 12 weeks of age and weighing around 300 to 400 g [28]. In Sprague Dawley rats, the bilateral CN is readily identifiable, running from the major pelvic ganglion (MPG) on the lateral side of the prostate, as a single individual nerve strand (see Figure 1B). In contrast, the corresponding structures in humans constitute the much more diffuse and convoluted autonomic nervous meshwork of the NVBs [14] (see Figure 1A). Thus, the CN lesion rat model is widely recognized as the go-to animal model for simulating post-RP ED in humans, primarily due to its anatomical advantages.

More specifically, due to the fact that, in male rats, the CN is anatomically isolated as a morphologically distinct entity within the pelvic cavity, as opposed to a complex plexus of autonomic nerves observed in humans, the model holds inherent anatomical suitability for CN dissection, manipulation, controlled lesions, and subsequent interventional therapeutic experimentation. Furthermore, from an experimental standpoint, this model additionally offers a reliable and objective quantification of nerve conduction and EF implicitly, obtained through corpus cavernosum cannulation, followed by ICP recordings. This allows for an empirical evaluation of CN lesion-induced conduction deficits and the functional outcomes of restorative interventions. Such evaluations are conducted through comparative analyses of alterations in ICP values, relative to MAP, achieved via standardized electrostimulation of the CNs, unilaterally or bilaterally, pre- and post-injury and/or therapeutic intervention [23].

Additionally, the economic viability of rat models adds another layer of practical advantage, adding to their appeal for research purposes, as the costs associated with the purchase, housing, and maintenance of rats are relatively low. Although mice may offer higher experimental throughput in certain cases, the rat model allows for the straightforward accomplishment of CN stimulation and ICP recording [29].

Overall, rats offer the advantage of convenient access to a wide array of behavioral, neurophysiological, molecular, biological, or genetic procedures, making them an excellent choice for multimodal investigations. However, there are limitations to consider. Most studies using this model have focused on young, healthy rats, which does not fully capture the clinical picture of PCa, which is more commonly diagnosed in older men, most with pre-existing conditions, such as diabetes, obesity, and cardiovascular issues. These conditions will impact EF independently, both in the preoperative setting, lowering baseline functionality, but also preoperatively, making the impact of post-RP CN damage a more complex topic in the real-life clinical PCa demographic. Conversely, especially in chronic studies, not allowing for a sufficient washout period for the therapeutics administered between stimulation response measurements may lead to a misinterpretation of EF recovery due to the continued presence and effects of experimental interventions [23].

Ironically, it was later-on demonstrated that, similarly to its advantages, the main limitation of the CN rat model is also anatomical. Importantly, the rat has been reported to possess additional ancillary penile innervation originating from the MPG, contributing to approximately 45% of ICP responses to supraspinal stimuli following CN transection [30]. Importantly, these ancillary nerves remain undamaged in the rat model of RP. The preservation of these nerve fibers should be considered when designing experiments, as they represent a potential source of bias. In fact, it has been shown that healthy rats will naturally recover EF over time (~6 weeks), even after bilateral CN injury [31], complicating the interpretation of therapeutic efficacy. However, it is worth mentioning that there are currently very few studies that have investigated the long-term impact of these ancillary nerves on EF recovery in rats.

## 3. Ethical, Methodological, and Technical Prerequisites

Rodents, particularly mice and rats, remain the favored choice for scientific research due to their small size, cost-effectiveness, rapid maturation, and genetic adaptability. In 2019, global scientific research involving animals reached an estimated annual total of 192.1 million animals [32]. Within Europe, including Norway, approximately 6.4 million mice and rats were utilized in 2018, constituting about 62% of animals employed across European Union (EU) member states. Consequently, the collective use of rats and mice worldwide likely exceeds 100 million annually [33].

To enhance the value of research involving animals, it is crucial to underscore the importance of adhering to the ARRIVE (Animal Research Reporting In Vivo Experiments) guidelines and recommendations [34]. The ARRIVE guidelines have been specifically crafted to elevate reporting standards and ensure that data stemming from animal experiments can undergo comprehensive evaluation and effective utilization. These guidelines encompass a 20-point checklist that outlines the essential information required in publications reporting on animal research.

This emphasis is particularly significant because, akin to humans, several mammalian species manifest signs of ED and alterations in vital regulatory pathways governing erection due to aging or various diseases [35]. Insufficient inclusion of critical information in research publications can hinder the accurate assessment of the final results obtained. Therefore, researchers are strongly encouraged to complete and submit the ARRIVE checklist alongside their manuscripts, ensuring transparency and comprehensibility in conveying their findings from animal research.

In the realm of conducting research involving rats, a meticulous approach is paramount to ensure both scientific integrity and the humane treatment of these animals. It all begins with obtaining the mandatory, primordial institutional approval and ethical clearance, often facilitated through channels such as the Institutional Animal Care and Use Committee (IACUC) in the United States of America (USA) or a similar regional pertinent ethics board. Afterward, careful attention is directed to the procurement and housing of the rats. This entails sourcing them from accredited suppliers or breeding facilities. The Sprague Dawley rat at around 10 to 12 weeks of age is recommended for acute CN injury studies. Also, providing them with a conducive environment, including maintaining ambient temperature between 20 °C and 24 °C (68°F–75°F), sustaining relative humidity between 40% and 60%, and adhering to a 12 h light/dark cycle to mimic natural circadian rhythms, is mandatory. Additionally, the rats are provided with a nutritionally balanced diet suitable for their age and research objectives, alongside unfettered access to uncontaminated drinking water [36].

The complex, invasive surgical procedures involved in CN injury rat model experiments necessitate the use of proper anesthetic methods in order to ensure that they can be carried out effectively. The role of anesthesia extends beyond mere pain relief for laboratory animals; it is also crucial for restraining the animal and reducing physiological changes that may arise from the animal’s stress response. The selection of anesthetics is pivotal, as some can profoundly influence physiological processes, such as those causing cardiorespiratory depression, which in turn can impact the outcomes of experimental research [37]. Therefore, the choice of anesthetic should be such that it does not induce significant alterations in the parameters under study. General anesthesia can be administered through inhalation or injection. The use of inhalation isoflurane anesthesia, offering superior anesthetic control and hemodynamic stability, is strongly advocated.

In microsurgery research, a low-magnification surgical microscope or loupes is crucial for clear field visualization. Either flip-up loupes or through-the-lens loupe models—the latter being specially customized for individual pupillary distance—with a magnification of 3X, up to 3.5X, are generally recommended. A microsurgery kit with electrocautery tools is also necessary. To stimulate the CN accurately, an electric stimulator with a 3 mm-spaced two-prawn electrode is ideal. Properly calibrated pressure transducers and recording equipment are vital for accurate documentation of MAP and ICP variations, ensuring valid, dependable results [38].

Finally, regulations mandate humane euthanasia upon the conclusion of a study or breeding program and when humane endpoints are met. Various international guidelines outline acceptable euthanasia methods for laboratory rodents, encompassing anesthesia overdose (via inhalation or injection), blunt force trauma, cervical dislocation, decapitation, exposure to carbon dioxide (CO_2_) or carbon monoxide (CO), and microwave irradiation. When animals are unconscious (e.g., under anesthesia), approved methods include exsanguination, air embolism, and the injection of substances like potassium chloride or ethanol. In EU member states and the United Kingdom (UK), the euthanasia of laboratory rodents is regulated by law, whereas in the USA, Canada, Australia, and New Zealand, regulations are less strict, with euthanasia methods governed by national guidelines and local policies, classified based on their capacity to provide a “humane” death [39].

## 4. Surgical Strategy Protocols

Before beginning any surgical preparation procedure, the rat will be administered general anesthesia and securely positioned. Achieving general anesthesia can be accomplished through the administration of inhaled or injectable anesthetics. In the case of inhaled anesthetics, a recommended approach involves using isoflurane (at concentrations of 3–5%) or sevoflurane (at concentrations of 2–4%) in combination with either oxygen or a mixture of oxygen and air [40]. These methods are commonly employed to induce general anesthesia effectively. In fact, the use of inhalation isoflurane anesthesia is strongly advocated as it allows for superior anesthetic control, particularly regarding the maintenance of stable hemodynamic parameters, such as blood pressure. However, it may also be advisable to use inhalational anesthesia as an induction step, having the test subject enclosed in a sealed container while the air mixture is being administered. Following this procedure, each animal may receive additional subcutaneous injections of fentanyl (235 mg/kg), fluanisone (7.5 mg/kg), and midazolam (3.75 mg/kg) [38,41,42].

**Figure 2:**
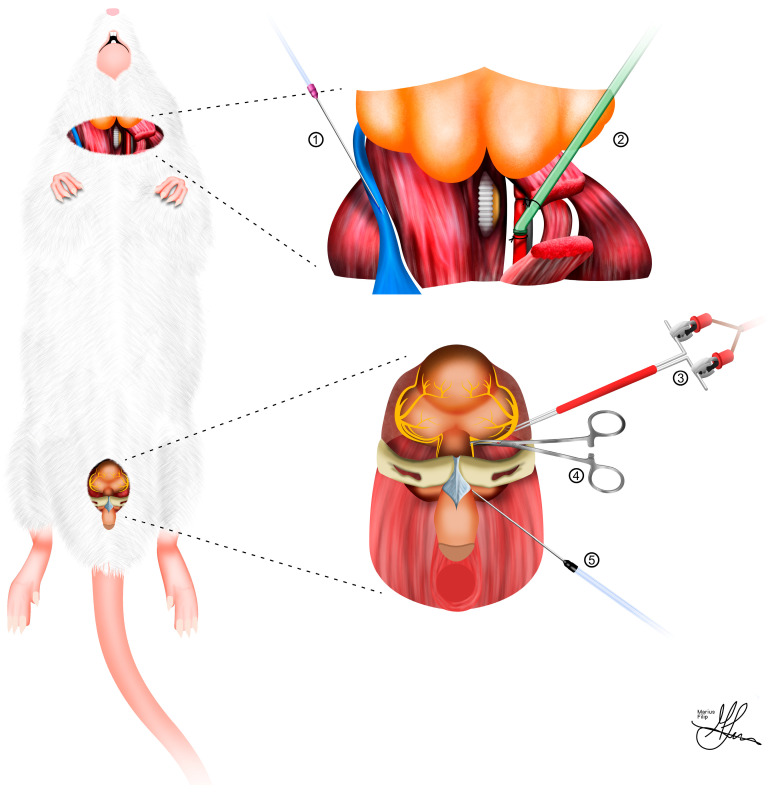
Illustration of an ideal cavernous nerve injury rat model experimental set-up: (**1**) jugular venous access; (**2**) carotid artery cannulation; (**3**) cavernous nerve electrostimulation; (**4**) cavernous nerve injury; (**5**) intra-cavernosal pressure measurement.

For injectable anesthetics, the current trend is towards modern combination medications [43], using intramuscular (IM) or intraperitoneal (IP) administration. These include a blend of tiletamine and zolazepam (Zoletil 100, 20–40 mg/kg, IM or IP), which can be used alongside xylazine (5–10 mg/kg, IM or IP). When used simultaneously, the dosage of each drug is lowered to the aforementioned minimum [42]. Subsequently, the neck and lower abdomen should be shaved and cleaned with 70% alcohol. The rat must then be secured in a supine position (see Figure 2) on a heated surgical pad or fastened to a test tube rack, and oxygen must be supplied through a nose cone at a constant rate of 0.8 L/min. Finally, the depth of anesthesia may be tested with a toe pinch.

In order to fully harness the extensive experimental potential of the CN injury rat model, the surgical strategies involved in the anatomical preparation of key topographic regions are multifaceted and warrant comprehensive preoperative evaluation. Typically, as shown in Figure 2, bilateral dissection of the CNs for injury and electrostimulation, along with cannulation of the carotid artery and corpus cavernosum, for MAP and ICP measurements, respectively, are integral components for achieving experimental accuracy [38]. In terms of standardizing CN surgical injuries, the crush lesion model is generally favored for systemic and topical intraoperative treatment studies, as opposed to the more radical cut lesion models, which necessitate subsequent micro-neurorraphy for repair [23].

### 4.1. Mean Arterial Pressure Measurements

For the precise measurement of MAP, surgical preparation should commence with a 1.5 cm transverse incision at the neck level, positioned precisely midway between the mandible and sternum. Caution must be exercised to avoid any disruption of superficial veins, and if necessary, cautery can be applied to manage any potential bleeding. Moving forward, throughout the surgery, the wound is irrigated with normal saline to prevent tissue desiccation [23,38].

**Figure 3:**
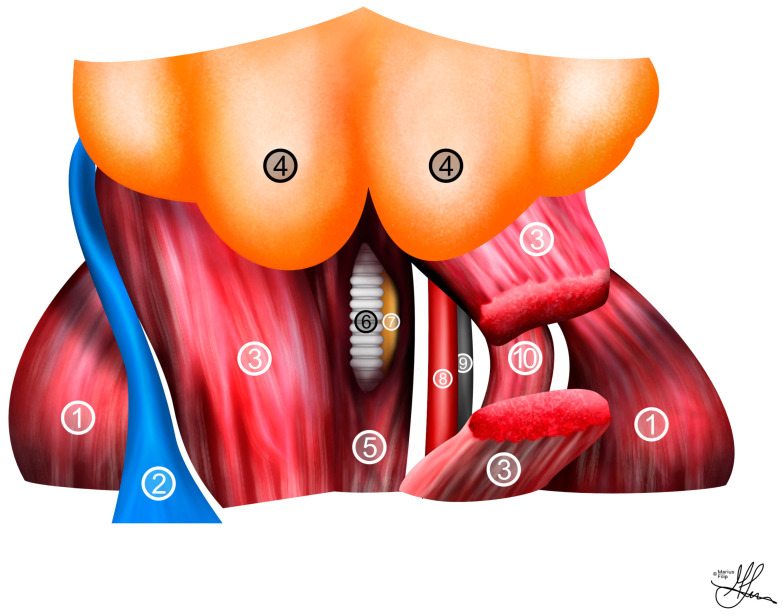
Illustration of neck anatomy in rats, coronal view: (**1**) cleidotrapezius muscle; (**2**) external jugular vein; (**3**) sternomastoid muscle; (**4**) submandibular salivary gland; (**5**) sternohyoid muscle; (**6**) trachea; (**7**) esophagus; (**8**) common carotid artery; (**9**) vagus nerve; (**10**) sternocleidomastoidian muscle.

Subsequently, an incision beneath the submandibular glands within the platysma muscle is created, carefully developing a space beneath this muscular layer to prevent additional superficial venous bleeding, which, even if it occurs, can also usually be safely controlled using cautery. Progress is thereafter made to reveal the strap muscles of the neck, followed by an incision in the sternohyoid muscle situated over the trachea (see Figure 3). This incision grants access to the pulsatile sheath enveloping the carotid artery. It is crucial to perform this dissection meticulously to avoid unintentionally involving the vagus nerve in the ensuing ligatures (see Figure 3), as this can lead to significant hemodynamic consequences [23,38]. Moreover, proper isolation from surrounding tissues and preparation of the carotid artery must be conducted attentively to ensure successful catheter implantation (see Figure 2).

**Figure 4:**
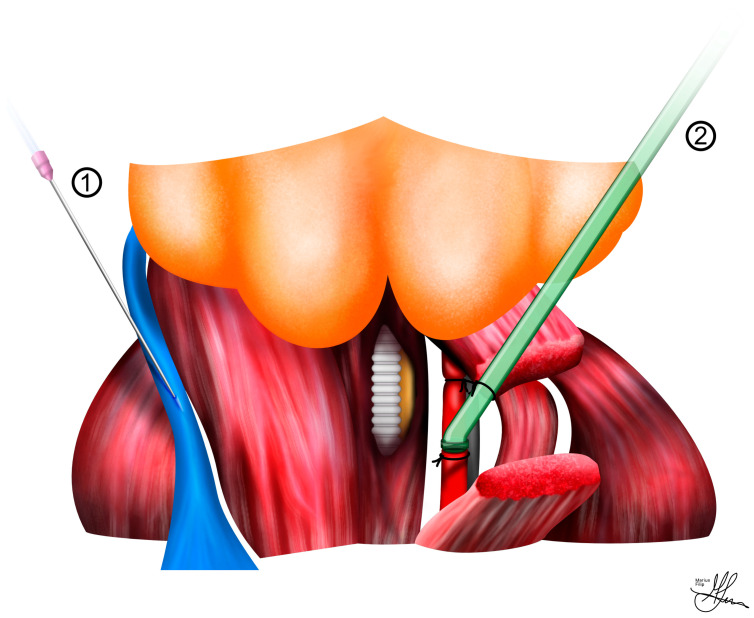
Illustration of (**1**) jugular venous access and (**2**) carotid artery cannulation for mean arterial pressure measurements in the cavernous nerve injury rat model—coronal view.

The carotid artery should be ligated superiorly, gently stretched, and secured with a second tie, which can also serve to anchor the tubing. Alternatively, when not utilizing a second tie, you may opt to secure the tubing with 4-0 silk sutures. Conclude by placing a bulldog clamp inferiorly and performing an arteriotomy. This arteriotomy provides the entry point for inserting a heparinized polyethylene tube using a 25-gauge needle with a bent tip (see Figure 4). Before initiating monitoring, ensure that the pressure transducer is zeroed at the level of the artery. This meticulous procedure guarantees the precise and dependable measurement of MAP, allowing for accurate monitoring throughout the surgical procedure.

When selecting a catheter for arterial cannulation, several key principles must be considered. Firstly, it is essential to choose a catheter model designed specifically for the relevant animal species and type of blood vessel. Secondly, compatibility with the external device it connects to is crucial. Lastly, the catheter’s design should be versatile and straightforward, accommodating a wide range of surgical procedures and research requirements. Rigid catheters remain a common choice for acute experiments due to their ease of implantation. However, it is worth noting the advantages of polyurethane, a thermoplastic polymer that softens within the vessel due to body heat. Its enhanced durability, compared to silicone, allows for thinner catheter walls and larger inner diameters, reducing the risk of catheter lumen blockage due to thrombus formation [42].

To maintain catheter patency and prevent occlusion, regular flushing is essential, with recommended intervals of at least every 30 min. Research indicates that intermittent, or bolus, flushing methods are significantly more effective at removing solid sediments from catheter walls compared to continuous flow. The most efficient approach involves delivering 2–3 consecutive boluses at 0.4-s intervals to create turbulent flow. It is important to exercise caution during fluid introduction into the catheter to avoid any potential damage to the vascular endothelium, as very abrupt fluid administration can have adverse effects [44].

### 4.2. Intra-Cavernosal Pressure Measurements

**Figure 5:**
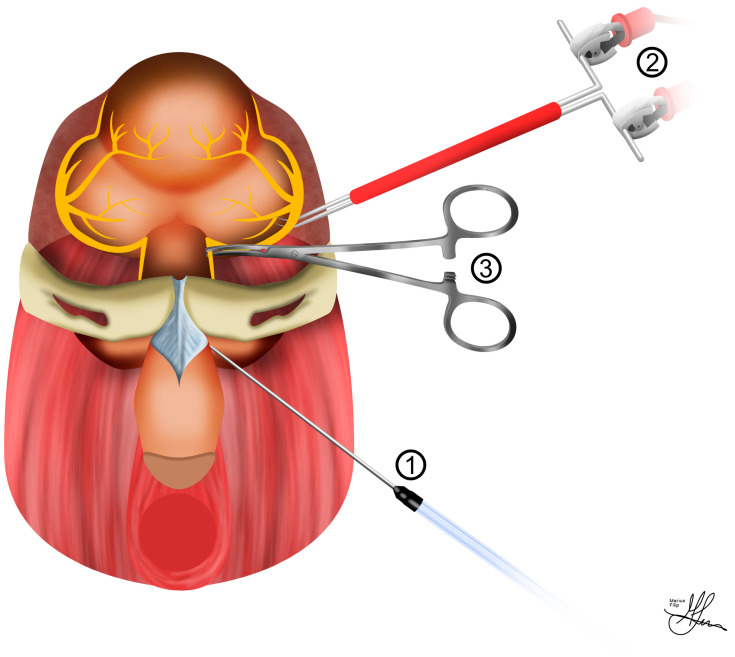
Illustration of pelvic preparation and essential procedures in the cavernous nerve (CN) rat model, coronal view: (**1**) Intra-cavernosal pressure measurement; (**2**) CN electrostimulation; (**3**) CN injury.

To prepare for ICP measurements, the penis is initially circumcised, its skin is carefully removed, and it is gently separated from the surrounding tissue to create a dedicated space around the bulbospongiosus muscle. For access, a precise 1 cm vertical skin incision is made, starting at the base of the penis, extending downward, and positioned 5 mm lateral to the midline. The use of a self-retaining retractor is highly beneficial at this stage, aiding in maintaining a clear surgical field. Given the relatively small diameter of the penis, ensuring accurate needle placement for ICP measurement is of utmost importance. The junction where the ischiocavernous muscle joins the inferior pubic rami is a distinct and recognizable landmark. The ischiocavernosus muscle is then exposed through careful blunt dissection, with Q-tips used for precision. Using curved microforceps, the muscle fibers are gently spread longitudinally, revealing the bright white tunica albuginea. Subsequently, a 21-gauge needle is carefully inserted into the corporal body to facilitate penile pressure measurements. Given the diminutive size, it is sufficient to insert only the bevel of the needle into the corporal body (see Figure 5). To ensure precise placement, a small quantity of heparinized saline can be flushed through the line. The correct placement of the needle is confirmed by observing slight penile tumescence [23,38]. To account for potential hemodynamic influences on ICP, it is strongly advisable to adjust ICP using MAP and report it as ICP/MAP, particularly when vasoactive substances are administered. As a good practice, consider including representative traces of both ICP and MAP in the manuscript. This inclusion allows readers and reviewers the opportunity to evaluate the quality of data registration and stimulation.

### 4.3. Cavernous Nerve Preparation

**Figure 6:**
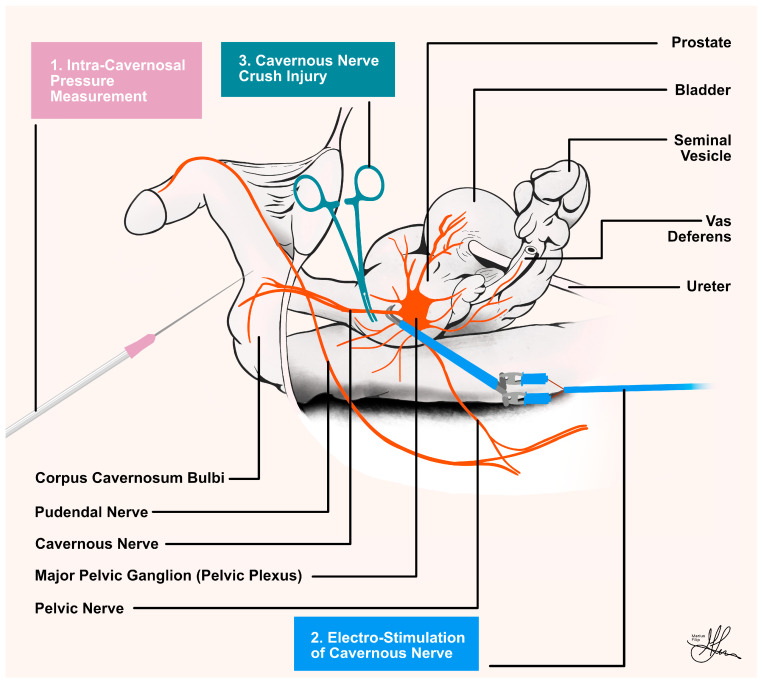
Illustration of pelvic preparation and essential procedures in the cavernous nerve (CN) rat model, sagittal view.

To access the CNs, a 2 cm vertical midline abdominal incision is made using a scalpel. After dissecting through the abdominal wall, the bladder is identified, decompressed, and retracted laterally and cranially. Any adhesions to the abdominal wall are divided. The dissection advances caudally, and the bladder and prostate are mobilized laterally to expose the seminal vesicle and vas deferens (see Figure 1B). The vas deferens is then dissected and mobilized superiorly in order to allow visualization of the dorsal aspect of the prostate. The area between the vas deferens and the dorsal lobe of the prostate is then cleared of any overlying fatty tissue in order to expose the CN. Dissection using cotton-tip swabs (Q-tips) is employed to expose the MPG and the CN (see Figure 1B). Close to the prostate lie pelvic veins, which need to be pushed off the gland with care in order to avoid bleeding. Micro scissors are utilized to incise the fascia overlaying the nerve on each side, and a 9-0 nylon suture is slid underneath the 0.3 mm section of CN freed from the underlying tissue. The CN is then separated from the surrounding tissue around 3–4 mm distal from the MPG. A microneedle holder/hemostat is subsequently placed on the CN and clamped, inducing a crush injury by clamping the nerve for two minutes (see Figure 6) [23,38].

As seen in Figure 5 and Figure 6, for stimulation of the CN, an electrode is required. The CN is lifted, and a 125 μm silver bipolar electrode is placed underneath. A micromanipulator is employed to hold the electrode. After slightly elevating the nerve so that neither the nerve nor the electrode touches the surrounding tissue, the electrode is dried, and biocompatible silicone glue (Kwik-Sil, WPI, Sarasota, FL, USA) is applied. After keeping the nerve elevated for 2–3 min (the time necessary for the glue to dry), a secure contact between the electrodes and the nerve is achieved. The nerve is then returned to its normal position, requiring no further manipulation. At this point, the abdominal incision is filled with 0.9% saline [23,38].

### 4.4. Standardized Cavernous Nerve Injury

One possible explanation for the disparity between clinical and animal data lies in the variability of methodologies employed in basic science studies. The lack of consensus guidelines for the utilization of the bilateral nsRP rat model, also known as bilateral CN crush, transection, excision, dissection, freezing, electrocautery, and irradiation model, has resulted in the publication of studies with frequently equivocal results that are challenging to compare [45].

Both unilateral and bilateral CN injury models have been employed to investigate ED and are considered to simulate the condition in humans after RP. Various types of CN injury have been explored in rodent models, particularly in rats. These models are categorized based on the type and extent of the injury, encompassing techniques such as stretching, crushing, freezing, transecting, dissecting, and excising the CN, as well as unilateral versus bilateral CN injury. Crushing of the CNs is the most commonly used method to replicate the nerve injury occurring during the nsRP procedure. Conversely, transection and excision of the CNs are predominantly used to mimic RP without the nerve-sparing procedure. The crush injury model involves subjecting the CN to various mechanical compressions over varying durations. The compression can be induced using various instruments, such as forceps, hemostatic clamps, or bulldog clamps, whether serrated or not. It remains unclear whether the choice of instrument or the timing of the crush can yield any differences in the magnitude and consistency of induced ED. Transection injury entails the direct division of the CN, while excision CN injury involves the removal of a segment of the CN. These CN injury techniques can be applied either unilaterally or bilaterally.

Unilateral nerve injury models, designed to replicate unilateral nsRP, typically preserve a portion of the nerve supply, resulting in the partial maintenance of penile erections. Moreover, it is believed that compensatory nerve sprouting from the intact contralateral CN occurs in these unilateral injury models. As a result, the contralateral side is often used as a control in unilateral injury models; however, the presence of contralateral sprouting can complicate the interpretation of experimental results. For these reasons, bilateral CN injury is considered the standard model, as it avoids such confounding factors [46].

It is worth noting that ancillary nerves have been reported to contribute to over 50% of pro-erectile innervation in the rat penis. These ancillary nerves are not affected in the CN injury model, and it remains uncertain whether this might influence the severity of ED in rats compared to humans. Importantly, the CN injury model emulates a “perfect” bilateral intrafascial nerve-sparing procedure, which can be challenging to perform on all patients in the clinical setting [30].

### 4.5. Standardized Electrostimulation Parameters

To properly position the stimulating electrode, it is customary to employ curved forceps as a guiding tool. The electrode itself is strategically placed at the junction point where the CN intersects with the MPG (see Figure 6), situated proximal to the lesion site. The initial round of stimulation serves the crucial function of verifying the effectiveness of the lesion. This verification hinges on the observation of a lack of conduction, which is characterized by the absence of any increase in penile pressure.

Optimal or maximal ICP responses are typically observed within the range of 6 to 7.5 V or 1.5 mA and at frequencies ranging from 10 to 20 Hz. It is considered ideal to generate voltage–response, intensity–response, or frequency–response curves to depict erectile responses under various electrical stimulation parameters. Additionally, another parameter of interest in nerve stimulation is the width or duration of the single pulse, which helps differentiate the threshold for activation of different types of nerves. This parameter has been reported to vary from 0.05 to 5 ms in various studies [47,48].

In the pursuit of uniformity and standardized procedures, well-defined electrostimulation protocols have been established for rat models with cavernous nerve lesions. An illustrative example of such a protocol, endorsed by the European Society for Sexual Medicine, includes specific settings for optimal results. These settings consist of a pulse duration of 0.5–1 ms, a frequency of 10–20 Hz, a stimulation duration of 30–60 s, and a voltage range of 2.5–8 V [47].

## 5. Promising Results and Future Perspectives

Surgical procedures for prostate and colorectal cancer frequently lead to a substantial occurrence of ED attributed to CN injury [49,50]. Despite advancements in surgical techniques, including nerve-sparing approaches aimed at preserving sexual function, ED continues to be a significant complication following these operations [51]. Numerous preclinical investigations have suggested the potential of PDE5 inhibitors (PDE5Is) to mitigate penile cell apoptosis and aid in penile rehabilitation [52,53]. However, recent large-scale clinical trials have indicated that routine postoperative PDE5I administration following pelvic surgeries may not effectively prevent the continued decline in EF [54,55,56].

Conversely, separate research involving a rat model suffering from CN injury-induced ED found that intracavernous injection of mesenchymal stem cells (MSCs) significantly improved EF [57]. While the precise mechanism is still under investigation, emerging literature suggests that the regenerative effects of MSCs are primarily mediated through paracrine activity, involving the secretion of various bioactive substances [58,59]. In addition to the secretion of soluble factors, MSCs have been found to release a range of substances, such as proteins, lipids, and nucleic acids, via extracellular vesicles (EVs). Notably, among these EVs, exosomes stand out [60]. Exosomes, measuring 40 to 150 nm in diameter, are generated within multivesicular endosomes or multivesicular bodies. Recent investigations have demonstrated the effectiveness of MSC-derived exosomes (MSC-Exos) in various animal models, including those related to stroke [61], hind-limb ischemia [62], cutaneous wounds [63], and kidney diseases [64]. These findings shed light on the diverse mechanisms through which MSCs can influence their host environment.

In a specific study, male Sprague Dawley rats have been used to investigate how surgical procedures affect EF and penile tissue changes. The rats were divided into four groups, each with eight subjects, and underwent either CN injury or sham surgery. Following these operations, the rats received intracavernous injections—phosphate-buffered saline, MSCs, or MSC-Exos—with subsequent assessment of their EF through electrical CN stimulation. This research design yields valuable insights into the importance of surgical-assisted assessment of EF in a rat model [65].

Other recent research has explored the role of IL-6-mediated inflammatory responses in post-RP ED. Using a rat model mimicking RP-induced CN lesions, Yamashita and colleagues administered Tocilizumab, an anti-rat IL-6 antibody, perioperatively. They discovered increased acute-phase interleukin (IL)-6 expression in the MPG after CN injury. Inhibiting IL-6 bioactivity appeared to ameliorate ED following CN simple dissection, as shown by enhanced ICP response rates to stimulation. This suggests that mitigating excessive inflammatory responses in the acute phase may hold promise for improving post-nsRP ED outcomes. In a similar context, Albersen et al. utilized pentoxifylline, a phosphodiesterase inhibitor with multi-cytokine pathway regulation effects. They applied it to a rat model with CN crush injury to enhance EF recovery, support nerve regeneration, and preserve corpus cavernosum microarchitecture. These studies contribute to our understanding of potential therapeutic interventions for post-RP ED [23].

Another area of potential surgical interest consists of the study of neuregulins (NRGs) and their effect on CN regeneration. NRGs are versatile growth factors impacting cell survival, proliferation, differentiation, and organ development—specifically glial growth factor 2 (GGF2), which we will further discuss. They achieve their effects by engaging with the ErbB family of transmembrane receptor protein tyrosine kinases, setting off a cascade of intracellular signaling events [66]. The NRG-1 gene is pivotal in facilitating axon–glial communication during peripheral nervous system development. Additionally, this growth factor is gaining recognition for its neuroprotective and neurorestorative attributes in adulthood, potentially by mediating axon–Schwann cell signaling crucial for efficient nerve repair [67]. The potential of NRGs to protect nerves and maintain EF following RP positions them as promising candidates. In a 2015 study by Burnett and colleagues, systemic GGF2 treatment initiated before CN injury not only facilitated the recovery of EF in rats but also preserved unmyelinated nerve fibers in the injured CN. This compelling outcome underscores the need for additional clinical investigations into the utility of GGF2 as a neuroprotective therapy for various pelvic surgeries, including RP, which remains an unaddressed aspect of research [68].

## 6. Conclusions

A comprehensive grasp of the technical intricacies outlined in the rat surgery procedure remains fundamental to the progression of our knowledge pertaining to ED and the responses of penile tissues. This mastery not only equips researchers but also catalyzes the exploration of surgical interventions and prospective treatments. Ultimately, this scientific expertise paves the way for the development of therapies aimed at addressing conditions affecting EF.
